# Regional radiomics similarity networks (R2SNs) in the human brain: Reproducibility, small-world properties and a biological basis

**DOI:** 10.1162/netn_a_00200

**Published:** 2021-08-30

**Authors:** Kun Zhao, Qiang Zheng, Tongtong Che, Martin Dyrba, Qiongling Li, Yanhui Ding, Yuanjie Zheng, Yong Liu, Shuyu Li

**Affiliations:** School of Biological Science & Medical Engineering, Beijing Advanced Innovation Center for Biomedical Engineering, Beihang University, Beijing, China; School of Computer and Control Engineering, Yantai University, Yantai, China; School of Biological Science & Medical Engineering, Beijing Advanced Innovation Center for Biomedical Engineering, Beihang University, Beijing, China; German Center for Neurodegenerative Diseases (DZNE), Rostock, Germany; School of Biological Science & Medical Engineering, Beijing Advanced Innovation Center for Biomedical Engineering, Beihang University, Beijing, China; School of Information Science and Engineering, Shandong Normal University, Jinan, China; School of Information Science and Engineering, Shandong Normal University, Jinan, China; School of Artificial Intelligence, Beijing University of Posts and Telecommunications, Beijing, China; Brainnetome Center & National Laboratory of Pattern Recognition, Institute of Automation, Chinese Academy of Sciences, Beijing, China; University of Chinese Academy of Sciences, Chinese Academy of Sciences, Beijing, China; School of Biological Science & Medical Engineering, Beijing Advanced Innovation Center for Biomedical Engineering, Beihang University, Beijing, China

**Keywords:** Regional radiomics similarity networks, Reproducibility, Small-world, Biological basis

## Abstract

A structural covariance network (SCN) has been used successfully in structural magnetic resonance imaging (sMRI) studies. However, most SCNs have been constructed by a unitary marker that is insensitive for discriminating different disease phases. The aim of this study was to devise a novel regional radiomics similarity network (R2SN) that could provide more comprehensive information in morphological network analysis. R2SNs were constructed by computing the Pearson correlations between the radiomics features extracted from any pair of regions for each subject (AAL atlas). We further assessed the small-world property of R2SNs, and we evaluated the reproducibility in different datasets and through test-retest analysis. The relationships between the R2SNs and general intelligence/interregional coexpression of genes were also explored. R2SNs could be replicated in different datasets, regardless of the use of different feature subsets. R2SNs showed high reproducibility in the test-retest analysis (intraclass correlation coefficient > 0.7). In addition, the small-word property (σ > 2) and the high correlation between gene expression (*R* = 0.29, *p* < 0.001) and general intelligence were determined for R2SNs. Furthermore, the results have also been repeated in the Brainnetome atlas. R2SNs provide a novel, reliable, and biologically plausible method to understand human morphological covariance based on sMRI.

## Author Summary

Gray matter volume and cortical thickness are some of the most popular brain morphological measures of structural magnetic resonance imaging (sMRI). These patterns are important for understanding complex brain cognitive function. However, most of the studies typically analyze single/several anatomical regions independently without considering associations among brain regions. The structural covariance network (SCN) is often used to reconstruct the brain structural network from sMRI and is commonly used to measure the association between regions in the human brain with morphological similarity. However, most of the individual SCNs have been constructed by a unitary marker such as gray volume/cortical thickness with hyposensitivity. We develop a novel, reliable and biologically plausible brain network to understand human morphological covariance based on sMRI.

## INTRODUCTION

Structural magnetic resonance imaging (sMRI) plays an important role in neuroscience, including in evaluations of gray matter volume and cortical thickness, which are some of the most popular brain morphological measures. However, most of the studies typically analyze single/several anatomical regions independently without considering associations among brain regions (Alexander-Bloch et al., 2013; Pichet Binette et al., 2020), especially regarding which complex heterogeneous network patterns can be used to characterize the brain by supporting information transformation; these patterns are important for understanding complex brain cognitive function (Bullmore & Sporns, 2012; Alexander-Bloch et al., 2013). Specifically, the structural covariance network (SCN) is often used to reconstruct the brain structural network from sMRI based on the similarity of gray matter morphology (He et al., 2007; Tijms et al., 2012) and is commonly used to measure the association between interregions in the human brain with morphological similarity (He et al., 2007; Montembeault et al., 2012; Tijms et al., 2012; Montembeault et al., 2016; Zhang et al., 2017; Seidlitz et al., 2018; Kong et al., 2019; Spreng et al., 2019).

The SCNs typically consist of nodes and edges, representing the predefined brain regions and the statistical similarity between them based on predefined morphological markers such as volume or thickness (For a review, see Alexander-Bloch et al., 2013). Several methodologies have been introduced for the reconstruction of connectome maps based on sMRI either at the group level [such as independent component analysis (Guo et al., 2015; Pichet Binette et al., 2020) and seed-based technology (Zielinski et al., 2010)] or the individual level. Specifically, SCN based on cortical thickness (He et al., 2007, 2008) or gray matter volume (Yao et al., 2010) was well studied at the group level, whereas the abovementioned biomarkers were also developed to construct brain networks using single or multiple morphological features at the individual level (Tijms et al., 2012; Wee et al., 2013; Kong et al., 2014; Kim et al., 2016). All these methods have been used to investigate network alterations in brain-related diseases (Yao et al., 2010; Zheng et al., 2015; Bethlehem et al., 2017; Yu et al., 2018). Seidlitz and colleagues proposed a morphometric similarity network that captures cortical cytoarchitecture and is linked to individual cognitive performance (Seidlitz et al., 2018), and it has been applied to understand major depressive disorder (Li et al., 2021). Despite the progress in constructing different brain networks, a well-validated and widely accessible model for mapping the brain network architecture of anatomical brain regions in an individual human brain is needed. Radiomics is a powerful, robust method to extract more detailed information (Li et al., 2019), including intensity and texture features from each brain region (Parmar et al., 2015; Gillies et al., 2016; Chaddad et al., 2018). Texture analysis describes a variety of features that quantify the variation in the patterns of intensity, including some imperceptible information to the human visual system (Kassner & Thornhill, 2010). However, there is a literature gap regarding the construction of a radiomics-based similarity network, as well as the associated attributes, which could be a feasible anatomical topological mapping of the individual brain.

In this study, the first aim was to develop a novel regional radiomics similarity network (R2SN) approach. Building on this foundation, the second aim was to explore the reproducibility, small-world property, and biological basis of the R2SNs, including the relationship between R2SN indices and the coexpression of the gene or general fluid intelligence (gF) scores. The results confirmed that R2SNs provide a novel, robust, and biologically plausible model and a new perspective for understanding the human brain; therefore, R2SNs have great promise in further studies.

## MATERIALS AND METHODS

### Subjects

A total of 848 subjects from the Human Connectome Project (HCP, https://www.humanconnectome.org/study/hcp-young-adult/document/) were included in our study; all subjects were cognitively normal controls (NCs; age: 28.82 ± 3.68, sex (M/F): 371/477, fluid intelligence: 16.53 ± 4.86, Flanker inhibitory control and attention (FICA) test score: 112.41 ± 10.06). The HCP was established in 2009 with an overarching objective of studying human brain connectivity and its variability in healthy adults (Van Essen et al., 2012). All HCP subjects were evaluated by 3T MR scanners. In the HCP protocol, fluid intelligence was assessed using a form of Raven’s progressive matrices with 24 items. Detailed subject information can be found at https://www.humanconnectome.org/study/hcp-young-adult/document/1200-subjects-data-release and can also be found in a previous study (Van Essen et al., 2012).

### Data Preprocessing and Radiomics Feature Extraction

**Figure 1.**
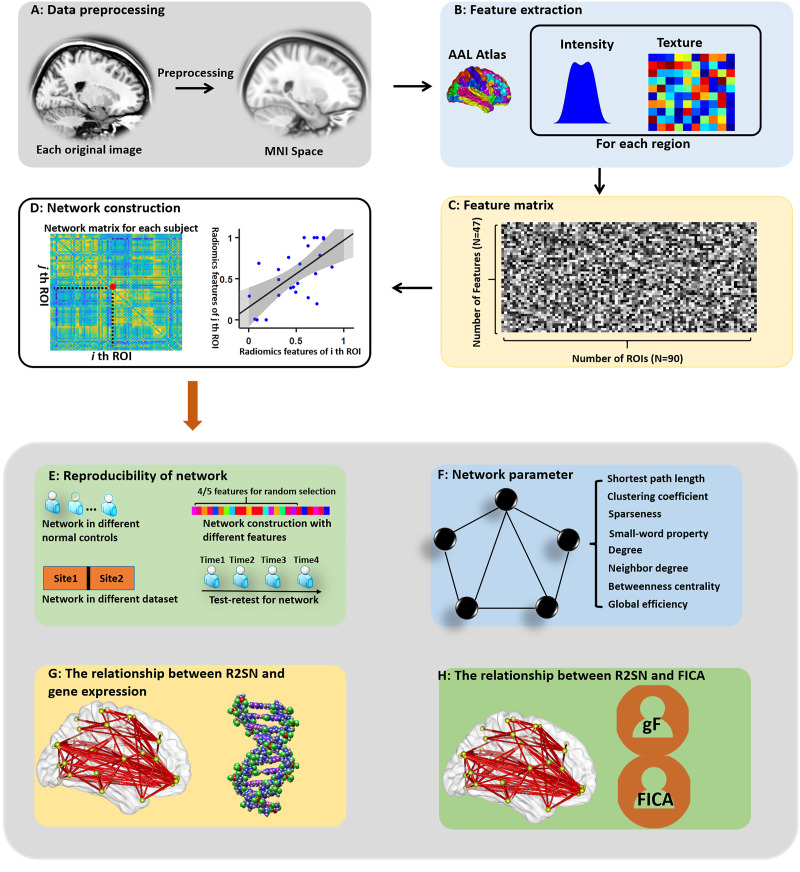
Schematic of the data analysis pipeline. (A) Data preprocessing. (B) The computation of radiomics features in each brain region. (C) Feature matrix of radiomics features. (D) Network construction with Pearson correlation. (E) The reproducibility of the network, (F) the network parameters of the R2SN, (G) the correlation between the R2SN and gene expression, and (H) the correlation between the R2SN and the gF score/FICA test score. gF indicates fluid intelligence; FICA indicates Flanker inhibitory control and attention.

For each subject, the T1-weighted MRI image was aligned to Montreal Neurological Institute (MNI) space by using a combined linear and nonlinear registration (including N4 bias field correction) and resampled to 1 mm × 1 mm × 1 mm (Xie et al., 2016) (Figure 1A). Then, 47 radiomics features in each brain region were extracted with each region defined in the AAL atlas (a total of 90 regions) (Tzourio-Mazoyer et al., 2002). The radiomics features consisted of 14 intensity features and 33 texture features (Figure 1B). All features were described in the study by Aerts et al. (2014) and implemented as in-house MATLAB scripts (https://github.com/YongLiulab/). The definitions and detailed descriptions of the radiomics features can be found in previous publications (Aerts et al., 2014; Feng et al., 2018; Zhao et al., 2020) and Supporting Information section S01. Redundancy features, defined as features having a high correlation with other features (*R* > 0.9), were removed before subsequent analysis (Supporting Information section S02). Therefore, a final feature matrix with 25 × 90 for each individual was obtained for further analysis (Figure 1C; Table 1).

**Table 1.** The reserved features after removing superfluous features

**Intensity features**	energy	**Textural features**	Autocorrelation	IMC1
	kurtosis		Cluster prominence	Maximum probability
	maximum		Cluster Shade	Sum entropy
	MAD		Cluster Tendency	Short run-length emphasis
	minimum		Contrast	Long run-length emphasis
	skewness		Correlation	Gray-level nonuniformity
	entropy		Energy	Low gray-level run-length emphasis
			Entropy	High gray-level run-length emphasis
			Homogeneity1	High gray-level long run-length emphasis

### R2SN Construction

An individual R2SN was constructed by feature normalization, followed by radiomics similarity matrix establishment. Specifically, feature normalization was implemented by adopting a common min-max feature normalization scheme, whereas the radiomics similarity matrix was established by mapping the individual’s radiomics features into a radiomics similarity matrix of pairwise interregional Pearson’s correlations (Figure 1D). Briefly, for each subject, a 90 × 90 connection matrix was obtained, the node of this network was defined as the region based on the AAL atlas, and the edge was calculated by Pearson’s correlations between interregional radiomics features (Figure 1D). The mean value and the standard deviation (Std) of the R2SNs were computed to estimate the fluctuation of R2SNs in these young NCs (Figure 1E).

### The Topological Structure of R2SNs

To explore the topological structure of the brain characterized by R2SNs, a variety of graph-theoretical network parameters were computed, including shortest path length (L), clustering coefficient (C), and small-world property, after binarization of R2SNs by using a threshold ranging from 0.5 to 0.75 (step size = 0.01). Briefly, the small-world index was defined as sigma (σ) = γ/λ, with gamma being (γ) = C_R2SN_/C_random_, lambda being (λ) = L_R2SN_/L_random_, and C_random_ and L_random_ being the C and L of the random network (random sampling of edges to yield a matrix with the same number of connections), respectively (Figure 1F). In addition, we computed the neighbor degree, degree, betweenness centrality, and global efficiency of the R2SNs by using a threshold ranging from 0.5 to 0.75 (step size = 0.01). The detailed definitions of these parameters can be found in a previous study (Rubinov & Sporns, 2010) and were computed by using the Brainnetome fMRI Toolkit (BRANT) (Xu et al., 2018) (Figure 1F).

### Reproducibility of the Network

We demonstrated three points of view to estimate the reproducibility of R2SNs, including the reproducibility of R2SN among different datasets, the reproducibility of the individual R2SN among different scans, and the robustness of R2SNs to methodological variations. First, the HCP dataset was randomly divided into two subdatasets for 1,000 iterations (424 subjects for each subdataset). The Pearson correlation coefficient of the two subdatasets in each iteration was used to estimate the consistency of the mR2SN (Seidlitz et al., 2018; Jin et al., 2020a; Zhao et al., 2020), which was defined as the mean value of the R2SNs in the specific dataset. Finally, the distribution of 1,000 Pearson correlation coefficients was used to estimate the reproducibility of the R2SNs (Seidlitz et al., 2018; Jin et al., 2020a; Zhao et al., 2020).

Notably, the test-retest analysis was used to estimate the reproducibility in different observers when measuring the same index (Friedman et al., 2008; Leijenaar et al., 2013; Maclaren et al., 2014; Noble et al., 2019). To investigate the reproducibility of the R2SN, a test-retest analysis was conducted using 21 subjects with four images acquired during different visits (https://duke.edu/∼morey005/ScanRescanData/). In this dataset, each subject was scanned on two different days, two scans were conducted 1 hr apart on day 1 (scans 1A and 1B), and two scans were conducted 1 hr apart at a second session 7–9 days later (scans 2A and 2B) (Morey et al., 2010). The intraclass correlation coefficient [ICC; ICC = (BMS − WMS)/BMS] was used to estimate the reproducibility of each edge of the R2SNs, where BMS is the between-subjects mean square and WMS is the within-subject mean square. The ICC has a value between 0 and 1; ICC = 0 indicates no reproducibility, and ICC = 1 indicates absolute reproducibility (Shrout & Fleiss, 1979) (Figure 1E).

We also quantified the robustness of R2SNs to methodological variations, including randomly reducing the number of radiomics features for analysis (i.e., only 20 radiomics features rather than all 25 features were involved as a predefined marker for network construction). The Pearson correlation coefficient was used to estimate the similarity of the mR2SN, which was constructed with 20 radiomics features and 25 radiomics features. The distribution of the Pearson correlation coefficient of 1,000 simulations was performed to assess the robustness of R2SNs to methodological variations (Seidlitz et al., 2018; Jin et al., 2020a; Zhao et al., 2020) (Figure 1E).

In addition, we explored whether a significant correlation could be obtained between the mean connective strength and the size of each node (the size of each node was roughly estimated with the voxel number based on the AAL atlas) (Tzourio-Mazoyer et al., 2002).

### The Relationship Between the R2SN and the Gene Similarity Network

To further explore the biological basis of R2SNs, we continued our investigation by computing the relationship between the R2SN and the gene similarity network (GSN). The GSN was constructed with the Allen atlas (https://human.brain-map.org/) (Zeng et al., 2012) and predefined genes (six subjects). The nodes were defined as the Allen atlas and mapped to the AAL brain regions based on MNI coordinates by using “abagen” (https://github.com/rmarkello/abagen) (Arnatkeviciute et al., 2019), and the edges were computed by the Pearson coefficient between the gene expression of any pair of regions. To correct the spatial autocorrelation effect within R2SN and GSN, the Euclidean distance of each pair of brain regions was employed as a concomitant variable. Pearson’s correlation coefficient between the connectivities of the mR2SN and the connectivities of the mGSN (the mean value of the GSN) was calculated to assess the similarity between the R2SN and GSN (Figure 1G). Furthermore, Pearson’s correlation coefficient between the mean connection strengths of nodes of the mR2SN and mGSN was calculated to evaluate the similarity of these two networks.

### Association Between the Network Properties of the R2SN and Cognitive Differences

We have investigated the network properties, reproducibility, and biological basis of the R2SN, and we assumed that the individual network properties could represent the individual’s differences in cognitive ability. In the HCP project, fluid intelligence scores were obtained as an index for measuring the subjects’ intrinsic cognitive ability. In addition, the Flanker task measures both a participant’s attention and inhibitory control. Therefore, we focused on the associations of common gF and the FICA test score as the indices to estimate the cognitive difference among young NCs, similar to Seidlitz et al. (2018). On this basis, we performed Pearson’s correlation analysis between gF/FICA scores and connectivity strength and network parameters (the neighbor degree, degree, betweenness centrality, global efficiency, shortest path length, and clustering coefficients) of the individual’s network to explore the relationship between the R2SN and gF/FICA scores in the HCP dataset.

### The Replicability of Results in Brainnetome Atlas

To explore the replicability of the results in different atlases, we repeated the above analysis in the Brainnetome atlas (Fan et al., 2016). First, the R2SN was created based on the Brainnetome atlas (*N* = 246 without cerebellar), and then the reproducibility, small-world properties and the biological basis of R2SN were explored with the same pipeline.

## RESULTS

### R2SN: Edge Properties

**Figure 2.**
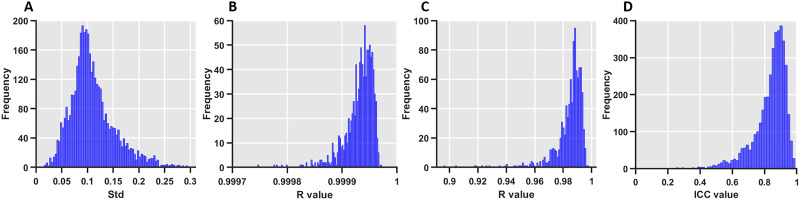
The reproducibility of the R2SN. (A) The distribution of variance in the HCP dataset. (B) The distribution of the correlation coefficients of the R2SN network for each pair of two datasets (the HCP dataset was divided into two parts for 1,000 random repetitions). (C) The distribution of the correlation coefficients of the R2SN network with 20 features (random selection of 20 features from 25 predefined features 1,000 times) and the R2SN created with 25 features. (D) The ICC value of the R2SN with test-retest analysis.

After redundancy removal, 25 radiomics features were reserved for each brain region, and the R2SN was constructed with those predefined features for each subject (a symmetrical matrix with a size of 90 × 90). The connective strength of the mR2SN ranged from −0.56 to 0.99 in the HCP dataset (*N* = 848), and the Std value in each edge of the R2SN was significantly smaller than the mean value, which ranged from 0.01 to 0.36, but with more than 95% of them ranging from 0.01 to 0.20 (Figure 2A).

### R2SN: Network Properties

**Figure 3.**
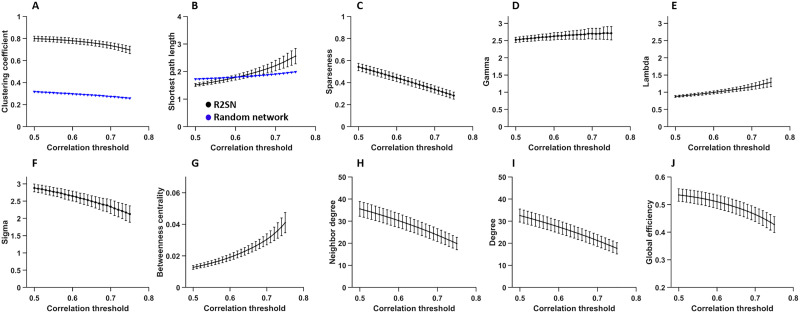
The network parameters of the R2SN with different correlation thresholds (0.5–0.75 and step size = 0.01). (A) The clustering coefficient—the “black” means the R2SN, and the “blue” means the random network; (B) the shortest path length; (C) sparseness, and the small-world parameter, including (D) the gamma value (the ratio of clustering coefficient between R2SN and random network); (E) the lambda value (the ratio of shortest path length between the R2SN and the random network); (F) the sigma value (the ratio of gamma and lambda); (G) the betweenness centrality value; (H) the neighbor degree value; (I) the degree value; and (J) the global efficiency value.

The C, L, and sparseness values are shown in Figure 3A–C. In addition, the lambda (λ) value showed the ratio of the L of the R2SN and the random network, and the gamma (γ) value showed the ratio of the C of the R2SN and the random network. As a result, the value of γ was significantly larger than 1 (Figure 3D), the value of λ was close to 1 (Figure 3E), and the small-world index sigma (σ) was also significantly larger than 1 by different thresholds of binarization (from 0.5 to 0.75 with a step size = 0.01) (Figure 3F). In addition, the betweenness centrality (Figure 3G), neighbor degree (Figure 3H), degree (Figure 3I) and global efficiency (Figure 3J) of the R2SNs with different thresholds (from 0.5 to 0.75 with a step size = 0.01) are also shown in Figure 3.

### Reproducibility of the R2SN

A series of hypothesis testing was performed to further assess the reproducibility of the R2SN. Most notably, high consistency was found in any two mR2SNs constructed by different datasets (1,000 random simulations), with the Pearson coefficient ranging from 0.9997 to 1 (Figure 2B). In addition, high consistency was also obtained by mR2SNs constructed with a different number of features (20 randomly selected features and all features), and the *R* value ranged from 0.879 to 0.997 (Figure 2C). More importantly, the R2SN had a high ICC value (ICC > 0.7) within more than a 95% edge by test-retest analysis (Figure 2D). We did not find a significant correlation between the size of the node and the mean connective strength (*R* = 0.14, *p* = 0.17).

### The Association Between the R2SN and the GSN

**Figure 4.**
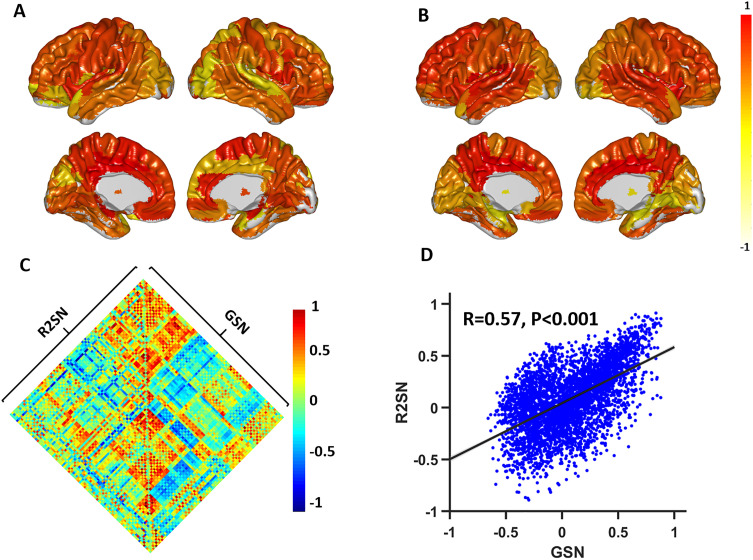
The correlation between the R2SN and the gene expression network. (A) The mean connective strength of the R2SN, which was mapped to the surface area from the AAL template. (B) The mean connective strength of the GSN, which was mapped to the surface area from the AAL template. The value of the color bar was normalized with the max-min method. (C) The heat map for the R2SN and GSN. Some negative correlations were generated with the GSN, and this phenomenon might have been caused by the deletion of genes in some brain regions. (D) The scatter diagram of the correlation between GSN and R2SN, where the Euclidean distance between each pair of regions of interest was employed as a concomitant variable within the network.

For each subject from the Allen dataset, the GSN was constructed based on 15,633 predefined genes (https://human.brain-map.org/). The mean connective strength of each node of the mR2SN and the mGSN are shown in Figure 4A (mR2SN) and Figure 4B (mGSN). We also computed the similarity between the mR2SN and the mGSN (edge-based), and a significant correlation was found between the two networks with *R* = 0.29 (*p* < 0.001) (Figure 4C and Figure 4D), meaning that the brain region with high morphometric similarity also tended to have a high transcriptional similarity of the gene. We also computed the average of the off-diagonal elements of a row or column in the radiomics similarity matrix (node-based), and a significant correlation was also found between the mR2SN and the mGSN regarding the mean connective strength of each node (*R* = 0.32, *p* = 0.002).

### The Association Between the R2SN and Cognitive Differences

**Figure 5.**
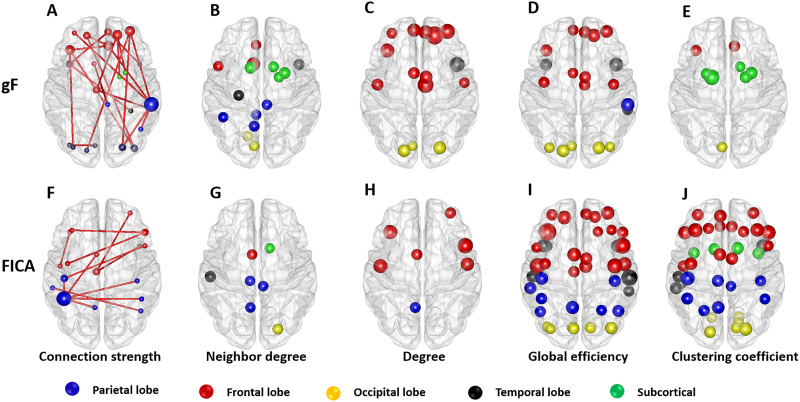
The correlation between the R2SN and cognitive ability. (A) Connectivity showed a significant correlation with the individuals’ gF score (Bonferroni-corrected *p* < 0.001); the brain regions in which the (B) neighbor degree value, (C) degree value, (D) global efficiency value, and (E) clustering coefficient showed a significant association with the gF score (Bonferroni-corrected *p* < 0.001). (F) Connectivity showed a significant correlation with the individuals’ FICA score (Bonferroni-corrected *p* < 0.001); the brain regions in which the (B) neighbor degree, (C) degree, (D) global efficiency, and (E) clustering coefficient showed a significant association with the FICA score (Bonferroni-corrected *p* < 0.001). FICA indicates the Flanker inhibitory control and attention test score, and gF indicates the fluid intelligence.

The fluid intelligence scores ranged from 4 to 24, and the FICA test scores ranged from 89 to 142 for all subjects (https://www.humanconnectome.org/study/hcp-young-adult). The Pearson’s correlation showed that approximately 5% of connections had a significant correlation with the fluid intelligence score (Bonferroni-corrected *p* < 0.05, with *N* = 4,005) (Figure 5A). As Figure 5 shows, the neighbor degree of 15 nodes (Figure 5B), the degree of 16 nodes (Figure 5C), the global efficiency of 20 nodes (Figure 5D), and the clustering coefficient of 8 nodes (Figure 5E) were significantly correlated with fluid intelligence (Bonferroni-corrected *p* < 0.001, with *N* = 90) (when the threshold = 0.50).

Pearson’s correlation showed that approximately 8% of connections had a significant correlation with the FICA test score (Bonferroni-corrected *p* < 0.05, with *N* = 4,005) (Figure 5F). In addition, the neighbor degree of 7 nodes (Figure 5G), degree of 7 nodes (Figure 5H), global efficiency of 42 nodes (Figure 5I), and clustering coefficient of 38 nodes (Figure 5J) were significantly correlated with the FICA test score (Bonferroni-corrected *p* < 0.001, with *N* = 90) (when the threshold is 0.50). The correlation between gF/FICA and the shortest path length and betweenness centrality is shown in Supporting Information section S03 due to the weaker significance.

### The Replicability of the Results in the Brainnetome Atlas

The results can be repeated in the Brainnetome atlas. First, high consistency was found in any two mR2SNs constructed by different datasets or subsets of the feature. More importantly, the R2SN had a high ICC value (ICC > 0.7) within more than 95% of edges by the test-retest analysis (Supporting Information Figure S1). Furthermore, the value of γ was significantly larger than 1, the value of λ was close to 1 and the small-world index sigma (σ) was also significantly larger than 1 by different thresholds of binarization (from 0.5 to 0.75 with a step size = 0.01) (Supporting Information Figure S2). In addition, the relationships between R2SN and gene expression (Supporting Information Figure S3)/fluid intelligence (Supporting Information Figure S4) were also verified in the Brainnetome atlas (Supporting Information S03).

## DISCUSSION

The present study provides a pipeline for transforming radiomics feature maps into a pairwise interregional radiomics similarity matrix at the individual level, which still represents a gap according to the literature. The systematic results confirmed that R2SNs provide a novel, robust, and biologically plausible model for understanding the human brain.

Radiomics is a powerful method to extract more detailed information from brain images, which includes intensity and texture features, and which could improve the ability of the SCN to represent the morphology of the brain as a network with the relativeness of pair of regions (Parmar et al., 2015; Gillies et al., 2016; Chaddad et al., 2018). Texture features can quantify the variations in intensity or patterns, including those features that are imperceptible to the human visual system (Aerts et al., 2014). Numerous studies have emphasized the importance of radiomics (Gillies et al., 2016) and have regarded it as a bridge between imaging and personalized medicine (Lambin et al., 2017). More importantly, radiomics also improve the diagnostic precision, treatment, and prognosis of a tumor (Aerts et al., 2014; Coroller et al., 2015; Huang et al., 2018) as well as the diagnosis of Alzheimer’s disease (Sorensen et al., 2016; Sorensen et al., 2017; Feng et al., 2018; Zhao et al., 2020; Ding et al., 2021). Radiomics is a method that extracts a large number of features of the brain region with high reproducibility (Li et al., 2019) and provides a better measure to evaluate the characteristics of the brain region.

Representing the morphology of the brain as a network has an advantage in that the structure of the brain can be described statistically with tools from graph theory (Tijms et al., 2012). Similar to most of the brain networks (e.g., functional brain networks, diffusion-weighted imaging, and SCNs) (He et al., 2007; Tijms et al., 2012; Heinze et al., 2015; Seidlitz et al., 2018), R2SNs also had complex topologies. In R2SNs, the high-degree nodes were located in the frontal lobe, parietal lobe, and occipital lobe, and low-degree nodes were found in the temporal lobe and subcortical lobe. The nodes with strong connection strengths might work more cooperatively with other brain regions, and the nodes with lower connection strengths might have a more specific function in the brain (Seidlitz et al., 2018). The different connective patterns between a GSN and R2SN might be caused by the difference in microstructure in the brain, such as the histological classification of cortical areas (Solari & Stoner, 2011; Seidlitz et al., 2018).

Notably, R2SN can be replicated in different datasets, demonstrated by a subsampling strategy from the same dataset for 1,000 iterations. R2SNs showed high ICCs (a prominent statistic to measure test-retest reliability) in different visit images, indicating the robustness of the radiomics similarity connectivity under the consensus that reproducibility was the most important property for a novel method in MRI analysis (Stikov et al., 2019). The high consistency of R2SNs, which was constructed with different features, also confirmed the methodological variations (Seidlitz et al., 2018). These properties of R2SNs support the firm foundation for the credibility of the results.

Imaging biomarkers are taken as the cornerstone of the radiology community, and imaging genetics has established heritable phenotypes for quantitative genetics of brain phenotypes. As expected, a high correlation was found between the gene expression network and the R2SN (Zeng et al., 2012), meaning that cortical areas with high morphometric similarity also tended to have high transcriptional similarity (Seidlitz et al., 2018). The structure of the brain region was controlled by gene expression (Warden & Mayfield, 2017), and the variations in intensity or pattern of the brain region can be reflected by radiomics features (Wang et al., 2019). These results further suggested that gene expression can be reflected by R2SNs. In brief, R2SNs have a genetic basis, and the present findings provide the possibility of estimating the risk of a gene by using R2SNs and indicate a certain degree of evidence for genetic disease research, such as that conducted regarding Alzheimer’s disease (Jin et al., 2020b; Zhao et al., 2020).

It is vital to evaluate the association between the individual network architecture and the cognitive ability or psychological functions of the brain (Li et al., 2009; van den Heuvel et al., 2009; Seidlitz et al., 2018). For high-degree hub nodes and the global efficiency of the connectome to be preferentially affected by clinical brain disorders associated with cognitive impairment, the relationship between high-degree nodes and cognitive status should obtain a higher performance in classification, prediction, and so on (Crossley et al., 2014). As we all know that fluid intelligence is a common measure to estimate the cognitive ability among NCs and to identify the cognitive difference among individuals, higher fluid intelligence scores corresponded to more efficient information transfer in the brain (Li et al., 2009). The fluid intelligence score refers to the ability to solve abstract problems that do not depend on acquired knowledge and change with age (Gray et al., 2003; Cole et al., 2012; Kievit et al., 2016). A set of fronto-parietal brain regions has been reported to be associated with fluid intelligence in brain imaging (Duncan et al., 2000; Cole et al., 2012; Woolgar et al., 2018). Comprehensive evidence has indicated that the lingual gyrus, caudate nucleus, rolandic operculum, and frontal lobe play an important role in an individual’s cognitive ability (Preusse et al., 2011; Rhein et al., 2014; Santarnecchi et al., 2015; Zhang et al., 2016). The significant correlation between fluid intelligence and efficiency of the hub regions in R2SNs also indicates that an individual’s cognitive ability is linked to the brain network architecture (Li et al., 2009; van den Heuvel et al., 2009). In addition, the network measures exclude global efficiency, which is also correlated with cognitive ability. Thus, we speculate that the R2SN might be a brain network with a solid biological basis.

This study has some limitations. First, we only studied R2SNs based on the AAL atlas and Brainnetome atlas; therefore, the network properties and their basis need to be further validated by other reputable brain atlases. Second, a unified framework for interpreting these measures and their alterations in different brain diseases is needed. The integration of existing studies shows that different models for brain structural networks do not align uniformly across the brain, and the coupling between structural network models and/or functional connectivity remodeling will help support the underpinnings of functional specialization and cognition. Third, more samples from different independent scanners and more cognition measures may improve the statistical power of the analysis, allowing scientists to explore the neural mechanisms of R2SNs in the future.

## CONCLUSION

R2SN is a network with high reproducibility and a biological basis; thus, an R2SN might serve as an improved, novel method and shed new light on future MRI studies. We assume that an R2SN could provide a powerful technology platform for measuring the anatomical connectome in vivo and be applied for the diagnosis of a variety of diseases in the future.

## ACKNOWLEDGMENTS

Data were provided by the Human Connectome Project, WU-Minn Consortium (Principal Investigators: David Van Essen and Kamil Ugurbil; 1U54MH091657) funded by the 16 National Institutes of Health institutes and centers that support the NIH Blueprint for Neuroscience Research and by the McDonnell Center for Systems Neuroscience at Washington University.

## AUTHOR CONTRIBUTIONS

Kun Zhao: Conceptualization; Data curation; Formal analysis; Investigation; Methodology; Visualization; Writing – original draft. Qiang Zheng: Funding acquisition; Investigation; Writing – review & editing. Tongtong Che: Writing – review & editing. Martin Dyrba: Writing – review & editing. Qiongling Li: Writing – review & editing. Yanhui Ding: Funding acquisition; Writing – review & editing. Yuanjie Zheng: Funding acquisition; Writing – review & editing. Yong Liu: Conceptualization; Funding acquisition; Writing – original draft; Writing – review & editing. Shuyu Li: Conceptualization; Funding acquisition; Writing – original draft; Writing – review & editing.

## SUPPORTING INFORMATION

Supporting information for this article is available at https://doi.org/10.1162/netn_a_00200. All subjects of this study are downloaded from the Human Connectome Project (HCP, https://www.humanconnectome.org/study/hcp-young-adult/document/), and the scripts of the radiomics features are available at https://github.com/YongLiulab.

## FUNDING INFORMATION

Shuyu Li, the National Natural Science Foundation of China, Award ID: 81972160, 81622025. Yong Liu, the National Natural Science Foundation of China, Award ID: 81871438, the Beijing Natural Science Funds for Distinguished Young Scholars, Award ID: JQ20036. Qiang Zheng, the National Natural Science Foundation of China, Award ID: 61802330. Yuanjie Zheng, the National Natural Science Foundation of China, Award ID: 81871508, 61773246, the Taishan Scholar Program of Shandong Province of China, Award ID: TSHW201502038. Yanhui Ding, the Natural Science Foundation of Shandong Province, Award ID: ZR2020MF051.

## TECHNICAL TERMS

Structural covariance network (SCN): A brain network from sMRI based on the similarity of gray matter morphology and is commonly used to measure the association between interregions in the human brain with morphological similarity.
Radiomics: A powerful, robust method to extract more detailed information from each brain region.
Small world network: Network with high clustering coefficient and short shortest path length.
Fluid intelligence: A measure which can reflect the cognitive ability.
Flanker inhibitory control and attention test: A measure which can reflect both a participant’s attention and inhibitory control.
Shortest path length: A shortest path between two nodes in a graph is a path with the minimum number of edge.
Sigma (σ): The small-world index was defined as sigma (σ) = γ/λ, with gamma being (γ) = C_R2SN_/C_random_, lambda being (λ) = L_R2SN_/L_random_, and with C_random_ and L_random_ being the C and L of the random network. C: clustering coefficient; L: shortest path length.
Random network: Network with low clustering coefficient and short shortest path length.
Global efficiency: The global efficiency is the average inverse shortest path length in the network.
Gene similarity network: A network based on the similarity of gene expression between each pair of brain regions.
