# Supplementary material for: Regional radiomics similarity networks (R2SNs) in the human brain: Reproducibility, small-world properties and a biological basis
Source: Netw Neurosci. 2021 Aug 30;5(3):783–97. doi: 10.1162/netn_a_00200 (PMC8567836; doi:10.1162/netn_a_00200)
Supplement: Supplementary file 1 [file netn-05-783-s001.pdf]

Zhao, K., Zheng, Q., Che, T., Martin, D., Li, Q., Ding, Y., Zheng, Y., Liu, Y. & Li, S. (2021). Supporting information for "Regional radiomics similarity networks (R2SNs) in the human brain: reproducibility, small-world properties and a biological basis." *Network Neuroscience.* Advance publication. https://doi.org/10.1162/netn_a_00200

Regional radiomics similarity networks (R2SN) in the human brain: reproducibility, small-world and biological basis

Outline

S01: Definitions of the radiomics features....2
1. Intensity features 2
2. Textural features 4
S02: Supplementary method for R2SN construction....7
S03: Supplementary results for the replicability of the result in Brainnetome Atlas 8
References....18

S01: Definitions of the radiomics features

A total of 47 MRI imaging features, including intensity and textural features, were extracted in this study (Aerts *et al.*, 2014). We provided this information in previous studies (Feng *et al.*, 2018; Zhao *et al.*, 2020). To maintain the integrity of this study, we also list it here.

1. Intensity features

Table S1. Intensity features describe the distribution of voxel intensities within an MRI image through commonly used and basic metrics. Because some intensity features have a close relationship with the voxel intensity, such as the maximum and mean, we first normalized those features by the image intensity using the min-max method in each center.

	Image feature	Equation	Definition
Intensity features (14)	Energy	$\sum_{i}^{N} X(i)^2$	Measure of the randomness of the intensity values in an image
	Entropy	$\sum_{i=1}^{N_l} P(i)\log_2 P(i)$	Represents the irregularity of the intensity value distribution
	Kurtosis	$\frac{\frac{1}{N}\sum_{i=1}^{N}(X(i)-\bar{X})^4}{\left(\sqrt{\frac{1}{N}\sum_{i=1}^{N}(X(i)-\bar{X})^2}\right)^2}$	The peakness of the histogram or indication of histogram flatness
	Maximum	Maximum intensity value of X	
	Mean	$\frac{1}{N}\sum_{i}^{N} X(i)$	Average intensity value of the pixels within the region of interest
	Mean Absolute Deviation (mad)	Mean of the absolute deviations of all voxel intensities around the mean intensity value	A measure of how much the gray levels differ from the mean

Median	Median intensity value of X	
Minimum	Minimum intensity value of X	
Range	Range of intensity values of X	
Root Mean Square (RMS)	$\sqrt{\frac{\sum_{i}^{N} X(i)^{2}}{N}}$	
Skewness	$\frac{\frac{1}{N}\sum_{i=1}^{N}(X(i)-\bar{X})^{3}}{\left(\sqrt{\frac{1}{N}\sum_{i=1}^{N}(X(i)-\bar{X})^{2}}\right)^{3}}$	Symmetry of intensity values in an image
Standard Deviation	$\left(\frac{1}{N-1}\sum_{i=1}^{N}(X(i)-\bar{X})^{2}\right)^{1/2}$	A measure of how much variation or dispersion exists
Uniformity	$\sum_{i=1}^{N_l} P(i)^{2}$	Measures the homogeneity of the intensity value distribution in an image
Variance (Var)	$\frac{1}{N-1}\sum_{i=1}^{N}(X(i)-\bar{X})^{2}$	The spread or variation around the mean (sum of squares)

X denotes the three-dimensional image matrix. ***N*** is the number of voxels. **P** is the first-order histogram with N_l discrete intensity levels. $\bar{X}$ is the mean of X. The number of histogram bins is 100.

2. Textural features

Table S2. Textural features describe the patterns or spatial distribution of voxel intensities.

	Image feature	Equation	Definition
	Autocorrelation	$\sum_{i=1}^{N_g}\sum_{j=1}^{N_g} ij\mathrm{P}(i,j)$	
	Cluster Prominence (CP)	$\sum_{i=1}^{N_g}\sum_{j=1}^{N_g}\left[i+j-\mu_x(i)-\mu_y(j)\right]^4\mathrm{P}(i,j)$	
	Cluster Shade	$\sum_{i=1}^{N_g}\sum_{j=1}^{N_g}\left[i+j-\mu_x(i)-\mu_y(j)\right]^3\mathrm{P}(i,j)$	
	Cluster Tendency	$\sum_{i=1}^{N_g}\sum_{j=1}^{N_g}\left[i+j-\mu_x(i)-\mu_y(j)\right]^2\mathrm{P}(i,j)$	
	Contrast	$\sum_{i=1}^{N_g}\sum_{j=1}^{N_g}\lvert i-j\rvert^2\,\mathrm{P}(i,j)$	Measures the local variation in intensity values
Textural features (33)	Correlation	$\frac{\sum_{i=1}^{N_g}\sum_{j=1}^{N_g} ijP(i,j)-\mu_i(i)\mu_j(j)}{\sigma_x(i)\sigma_y(j)}$	Measures the linear dependencies of intensity values in an image
	Difference Entropy	$\sum_{i=0}^{N_g-1} P_{x-y}(i)log_2\left[P_{x-y}(i)\right]$	
	Dissimilarity	$\sum_{i=1}^{N_g}\sum_{j=1}^{N_g}\lvert i-j\rvert\,\mathrm{P}(i,j)$	
	Energy	$\sum_{i=1}^{N_g}\sum_{j=1}^{N_g}[\mathrm{P}(i,j)]^2$	
	Entropy	$-\sum_{i=1}^{N_g}\sum_{j=1}^{N_g}\mathrm{P}(i,j)\,log_2[\mathrm{P}(i,j)]$	
	Homogeneity1	$\sum_{i=1}^{N_g}\sum_{j=1}^{N_g}\frac{\mathrm{P}(i,j)}{1+\lvert i-j\rvert}$	Measures the homogeneity of the intensity values

Homogeneity2	$\sum_{i=1}^{N_g}\sum_{j=1}^{N_g}\frac{\mathrm{P}(i,j)}{1+\lvert i-j\rvert^2}$	Measures the homogeneity of the intensity values of the pixel pair
Informational Measure of Correlation 1 (IMC1)	$\frac{HXY-HXY1}{max\{HX,HY\}}$	
Informational Measure of Correlation 2 (IMC2)	$\sqrt{1-e^{-2(HXY2-HXY)}}$	
Inverse Difference Moment Normalized (IDMN)	$\sum_{i=1}^{N_g}\sum_{j=1}^{N_g}\frac{\mathrm{P}(i,j)}{1+\left(\frac{\lvert i-j\rvert^2}{N^2}\right)}$	
Inverse Difference Normalized (IDN)	$\sum_{i=1}^{N_g}\sum_{j=1}^{N_g}\frac{\mathrm{P}(i,j)}{1+\left(\frac{\lvert i-j\rvert}{N}\right)}$	
Inverse Variance	$\sum_{i=1}^{N_g}\sum_{j=1}^{N_g}\frac{\mathrm{P}(i,j)}{\lvert i-j\rvert^2}, i\neq j$	
Maximum Probability	$\max\{P(i,j)\}$	
Sum Average	$\sum_{i=2}^{2N_g}\left[i\mathrm{P}_{x+y}(i)\right]$	
Sum Entropy	$-\sum_{i=2}^{2N_g}\mathrm{P}_{x+y}(i)log_2\left[\mathrm{P}_{x+y}(i)\right]$	
Sum Variance	$\sum_{i=2}^{2N_g}(i-SE)^2\,\mathrm{P}_{x+y}(i)$	
Variance	$\sum_{i=1}^{N_g}\sum_{j=1}^{N_g}(i-\mu)^2\,\mathrm{P}(i,j)$	
Short Run-Length Emphasis (SRE)	$\frac{\sum_{i=1}^{N_g}\sum_{j=1}^{N_r}\left[\frac{p(i,j\|\theta)}{j^2}\right]}{\sum_{i=1}^{N_g}\sum_{j=1}^{N_r}p(i,j\|\theta)}$	
Long Run-Length Emphasis (LRE)	$\frac{\sum_{i=1}^{N_g}\sum_{j=1}^{N_r}j^2\,p(i,j\|\theta)}{\sum_{i=1}^{N_g}\sum_{j=1}^{N_r}p(i,j\|\theta)}$	
Gray-Level Nonuniformity (GLN)	$\frac{\sum_{i=1}^{N_g}\left[\sum_{j=1}^{N_r}p(i,j\|\theta)\right]^2}{\sum_{i=1}^{N_g}\sum_{j=1}^{N_r}p(i,j\|\theta)}$	Represents the similarity of intensity values in an image

$\mathrm{P}(\mathrm{i},\mathrm{j})$ is the co-occurrence matrix for an arbitrary δ and α

N_g is the number of discrete intensity levels in the image

$p(i,j|\theta)$ is the (i,j)th entry in the given run-length matrix p for a direction θ

N_g is the number of discrete intensity values in the image

N_r is the number of different run lengths

N_p is the number of voxels in the image

u is the mean of $P(i,j)$

$p_x(i)=\sum_{j=1}^{N_g} P(i,j)$ is the marginal row probabilities

$p_y(i)=\sum_{i=1}^{N_g} P(i,j)$ is the marginal column probabilities

μ_x is the mean of p_x

μ_y is the mean of p_y

σ_x is the standard deviation of p_x

σ_y is the standard deviation of p_y

$p_{x+y}(k)=\sum_{i=1}^{N_g}\sum_{j=1}^{N_g} P(i,j)$, i+j=k, k=2,3,…,2*$N_g$

$P_{x-y}(k)=\sum_{i=1}^{N_g}\sum_{j=1}^{N_g} P(i,j)$,$|i-j|$=k, k=0,1,…,$N_g-1$

HX=$-\sum_{i=1}^{N_g} p_x(i)\log_2[p_x(i)]$ is the entropy of p_x

HY=$-\sum_{i=1}^{N_g} p_y(i)\log_2[p_y(i)]$ is the entropy of p_y

H=$-\sum_{i=1}^{N_g}\sum_{j=1}^{N_g} P(i,j)\log_2[P(i,j)]$ is the entropy of $P(i,j)$

HXY1=$-\sum_{i=1}^{N_g}\sum_{j=1}^{N_g} P(i,j)log\left(p_x(i)p_y(j)\right)$

HXY2=$-\sum_{i=1}^{N_g}\sum_{j=1}^{N_g} p_x(i)p_y(j)log\left(p_x(i)p_y(j)\right)$

S02: Supplementary method for R2SN construction

We first performed a common min-max method to normalize the radiomics feature among different brain regions in individuals, and the redundancy feature was defined as those features that had a high correlation with other features (R>0.9). Briefly, we computed the correlation between the i th (i=1.2…47) feature and the rest of the features, and the feature was redundant if an R value>0.9 appeared once or more. These superfluous features were removed before subsequent analysis. As a result, a final feature matrix of 25 × 90 for each subject was obtained for further analysis (Table1). The detailed steps of the R2SN construction are shown in Figure S1.

For each regions:
Feature matrix (M) with 90 Brain regions*47 Radiomics features
Feature matrix (M') with 90 Brain regions*25 Radiomics features
after removing redundant features
Net(i,j) = Correlation(M'(i,:), M'(j,:))
i=1,2…90; j=1,2…90

Figure S1. The detailed steps of the R2SN construction.

S03: Supplementary results for the replicability of the result in Brainnetome Atlas

Overall, a high consistency was found in any two mR2SNs that were constructed by different datasets (1000 times randomly simulation), and the Pearson coefficient ranged from 0.9997 to 1 (Figure S1b). In addition, a high consistency was also obtained by mR2SNs, which were constructed with different numbers of features (20 features for randomly selected and all features), and the R-value ranged from 0.90 to 1 (Figure S1c). More importantly, the R2SN achieved a high ICC value (ICC > 0.7) within more than 95% of edges by test-retest analysis (Figure S1d).

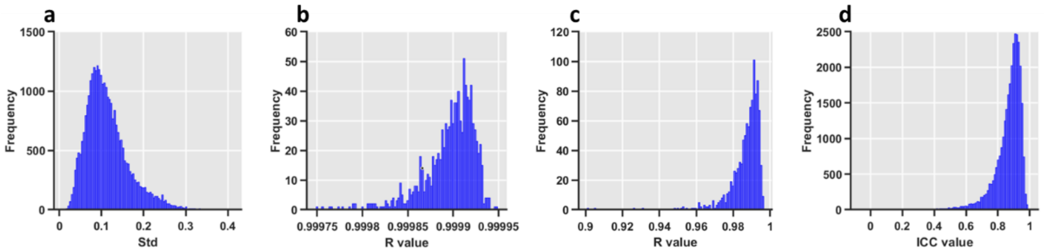

Figure S2. The stability and reliability of the R2SN. (a) The distribution of variance in the HCP dataset. (b) The distribution of the correlation coefficients of the R2SN network for each pair of two datasets (the HCP dataset was divided into 2 parts for 1000 random repetitions). (c) The distribution of the correlation coefficients of the R2SN network with 20 features (random selection of 20 features from 25 predefined features 1000 times) and the R2SN created with 25 features. (d) The ICC value of the R2SN with test-retest analysis.

The clustering coefficients, shortest path length, and sparseness are shown in Figure S2a-c. In addition, lambda (λ) indicates the ratio of the shortest path length of R2SN and the random network, and gamma (γ) indicates the ratio of the clustering coefficient of R2SN and the random network. As a result, the value of λ was close to 1 (Figure S2d), the value of γ was significantly larger than 1 (Figure S2e), and the small world index sigma (σ) was also significantly larger than 1 by different thresholds of binarization (from 0.5 to 0.75, and the step size =0.01) (Figure S2f).

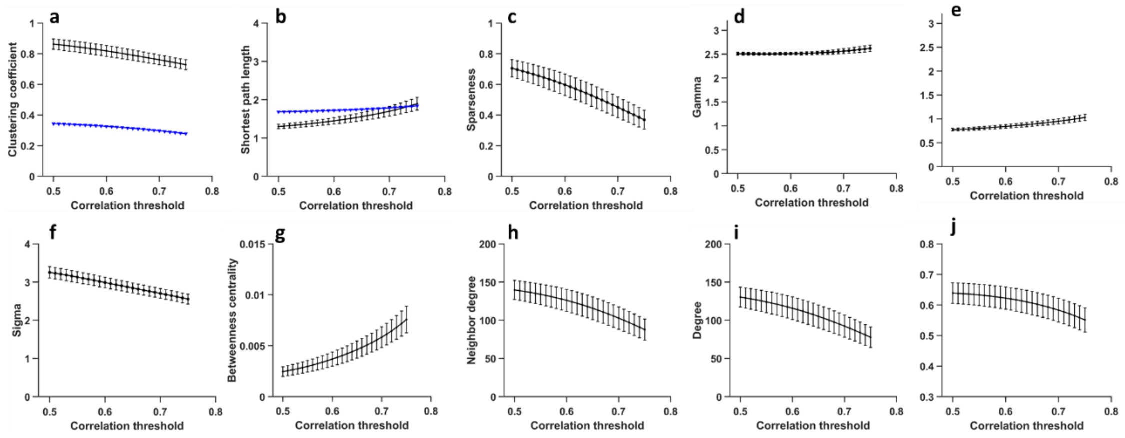

Figure S3. The network parameters of the R2SN with different correlation thresholds (0.5-0.75 and step size=0.01). (a) The clustering coefficient—"black" indicates the R2SN, and "blue" indicates the random network, (b) the shortest path length, (c) sparseness, and the small-world parameter, including (d) the gamma value (the ratio of clustering coefficients between R2SN and the random network), (e) the lambda value (the ratio of shortest path lengths between the R2SN and the random network), (f) the sigma value (the ratio of gamma and lambda), (g) the betweenness centrality value, (h) the neighbor degree value, (i) the degree value and (j) the global efficiency value.

To further explore the network structure, we also computed the hub nodes of the R2SN. In this study, the hub nodes were defined as those nodes that had many connections with other nodes. As Figure S4 shows, the hub nodes of the R2SN based on the AAL atlas were located mainly in the PrCG, SFGdor, ORBsup, MFG, ORBmid, IFGoperc, IFGtriang, SMA, CAL, CUN, SOG, PoCG, SMG, and HIP (Degree>10). Meanwhile, the hub nodes of the R2SN based on the BN atlas were located mainly in the A8m, A6m, A14m, A11m, A11m, A12_47l, A12_47l, A4tl, A6cvl, and A1_2_3tonLa. The PrCG, SFG, MFG, IFG and PoCG have been reported as the hub regions in previous studies (He *et al.*, 2007; Tijms *et al.*, 2012; Kim *et al.*, 2016).

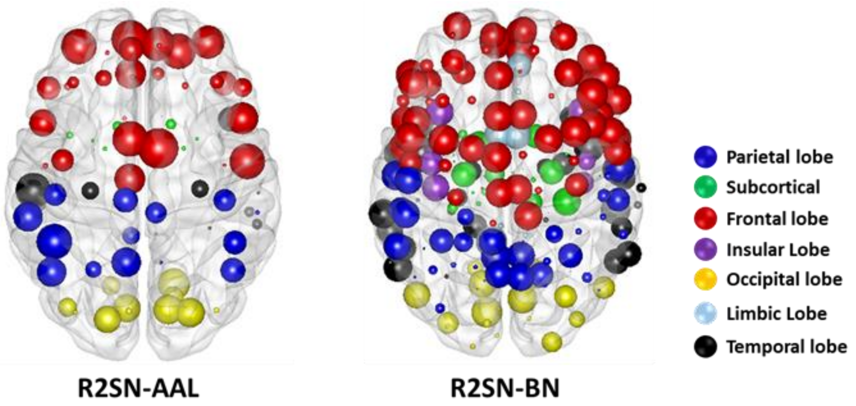

Figure S4. The hub nodes of the R2SN are based on the AAL atlas and BN atlas.

We also computed the correlation between the degree of the node and the cognitive score. PrCG, ORBsup, ORBmid, IFGoperc, SMA, CAL, CUN, SOG, PoCG, SMG, and HIP showed a significant correlation with the cognitive score. The hub regions are important for the connectome's efficiency and are preferentially affected by neurodegenerative disease (Seidlitz *et al.*, 2018). These hub regions require more attention in future studies.

The mean connective strengths of each node of mR2SN and mGSN are shown in Figure S5a (mR2SN), Figure S5b (mGSN), and Figure S5c. We also computed the similarity between mR2SN and mGSN (edge-based measure), and the Euclidean distance between each pair of ROIs was employed as a concomitant variable. A significant correlation can be found between the two networks with R=0.25 (P<0.001), meaning that the brain region with high morphometric similarity also tended to have a high transcriptional similarity of the gene.

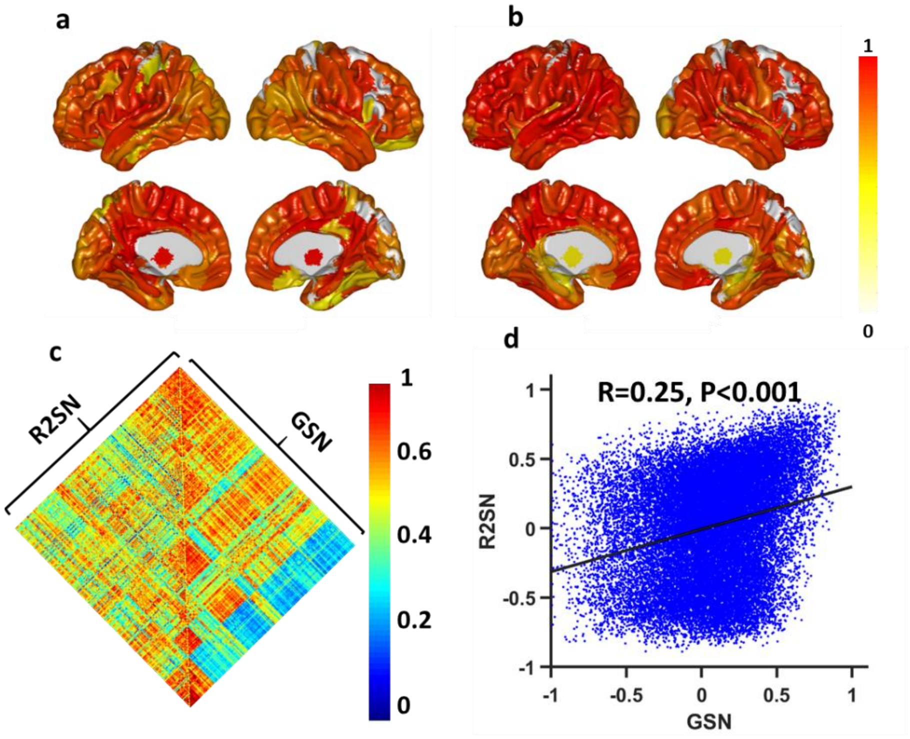

Figure S5. The correlation between the R2SN and the gene expression network. (a) The mean connective strength of the R2SN, which was mapped to the surface area from the Brainnetome Atlas. (b) The mean connective strength of the GSN, which was mapped to the surface area from the Brainnetome Atlas template. The value of the color bar was normalized with the max-min method. (c) The heat map for the R2SN and GSN. Some negative correlations were generated with the GSN, and this phenomenon might have been caused by the deletion of genes in some brain regions. (d) The scatter diagram of the correlation between GSN and R2SN, where the Euclidean distance between each pair of ROIs was employed as a concomitant variable within the network.

The Pearson correlation showed that approximately 1% of connections have a significant correlation with the fluid intelligence score (Bonferroni-corrected P<0.05, with N=30315) (Figure S6a). As Figure S6 shows, the neighbor degree of 7 nodes (Figure S6b), degree of 39 nodes (Figure S6c), global efficiency of 82 nodes (Figure S6d) and clustering coefficient of 145 nodes (Figure S6e) were significantly correlated with fluid intelligence (Bonferroni-corrected P<0.05, with N=246) (when threshold =0.50). In addition, the neighbor degree of 0 nodes, degree of 15 nodes, global efficiency of 23 nodes and clustering coefficient of 13 nodes were significantly correlated with fluid intelligence (Bonferroni-corrected P<0.01, with N=246) (when threshold =0.50).

The Pearson correlation showed that approximately 9% of connections have a significant correlation with fluid intelligence score (Bonferroni-corrected P<0.05, with N=30315) (Figure S6f). As Figure S6 shows, the neighbor degree of 59 nodes (Figure S6g), degree of 41 nodes (Figure S6h), global efficiency of 95 nodes (Figure S6i) and clustering coefficient of 198 nodes (Figure S6j) were significantly correlated with fluid intelligence (Bonferroni-corrected P<0.001, with N=246) (when threshold =0.50).

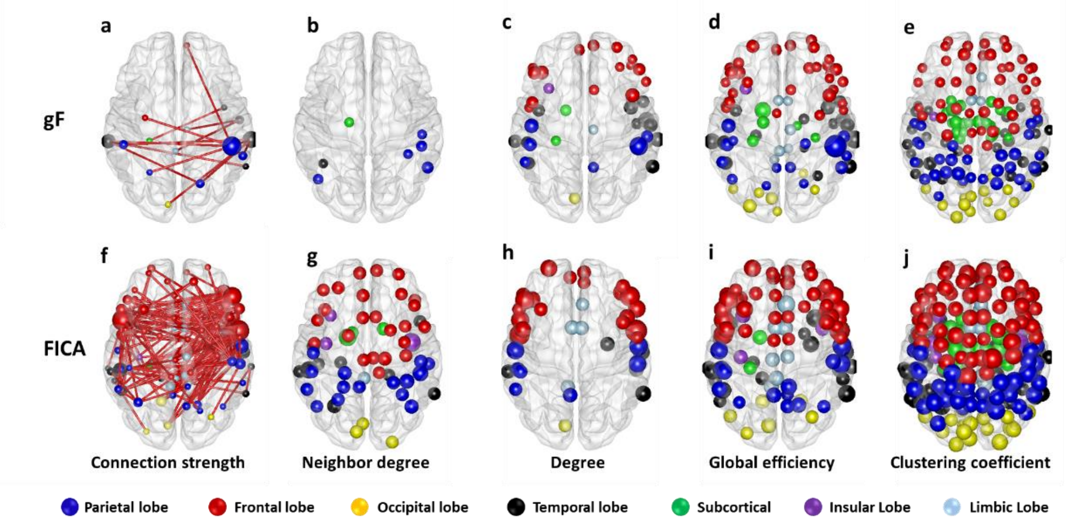

Figure S6. The correlation between R2SN and cognitive ability. (a) Connectivity showed a significant correlation with the individuals' fluid intelligence score (Bonferroni-corrected P<0.001), and the brain regions in which the (b) neighbor degree, (c) degree, (d) global efficiency and (e) clustering coefficient showed a significant association with the gF score (Bonferroni-corrected P<0.05). (f) Connectivity showed a significant correlation with the individuals' FICA score (Bonferroni-corrected P<0.05), and the brain regions in which the (b) neighbor degree, (c) degree, (d) global efficiency and (e) clustering coefficient showed a significant association with the FICA score (Bonferroni-corrected P<0.05).

FICA indicates the Flanker inhibitory control and attention test score, and gF indicates the fluid

intelligence.

We also computed the correlations between betweenness centrality/shortest path length and gF/FICA. However, weak significance was obtained as shown in Figure S7.

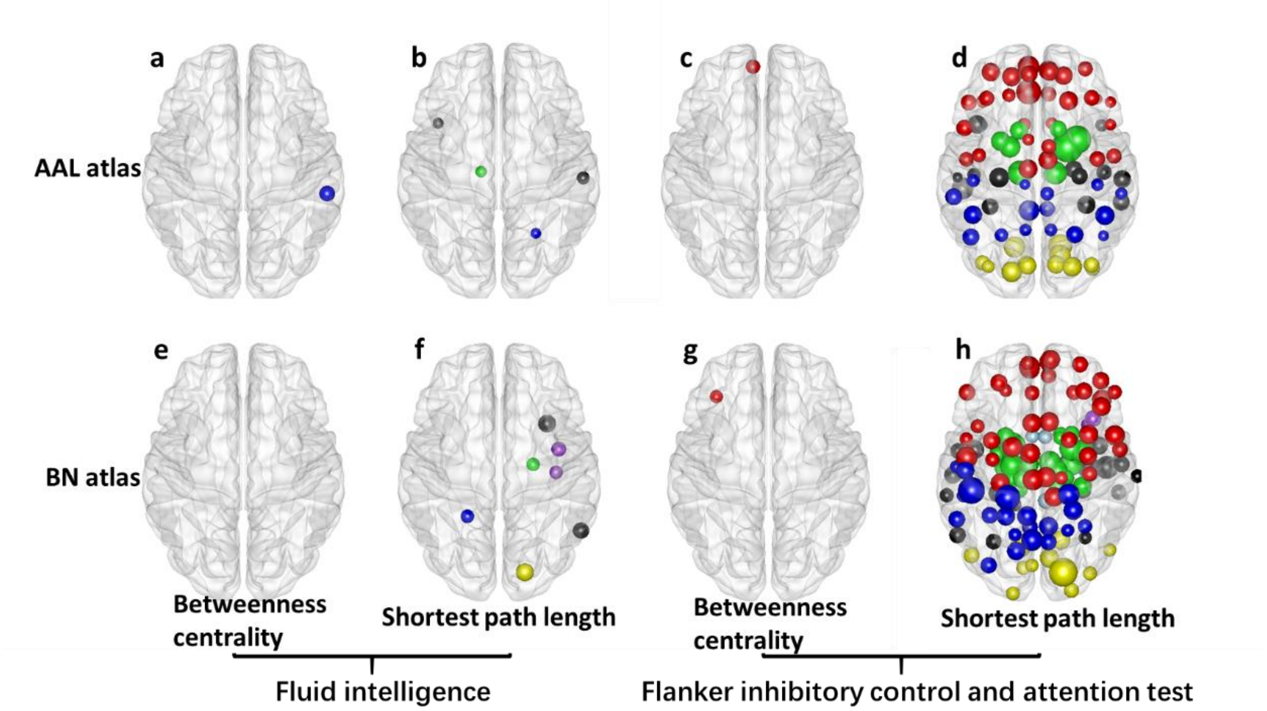

Figure S7. The correlation between R2SN and cognitive ability. The brain regions based on the AAL atlas in which (a) the between betweenness centrality and (b) the shortest path length showed a significant association with the gF score (Bonferroni-corrected P<0.05); the brain regions based on the AAL atlas in which (c) the between betweenness centrality and (d) the shortest path length showed a significant association with the FICA score (Bonferroni-corrected P<0.05). The brain regions based on the BN atlas in which (e) the between betweenness centrality and (f) the shortest path length showed a significant association with the gF score (Bonferroni-corrected P<0.05); and the brain regions based on the BN atlas in which (g) the between betweenness centrality and (h) the shortest path length showed a significant association with the FICA score (Bonferroni-corrected P<0.05).

S04: Brain region names

Table S3. The detailed brain region names of the AAL atlas (https://www.gin.cnrs.fr/en/tools/aal/).

ID	Label	Description	ID	Label	Description
1	PreCG_L	Precentral_L	46	CUN_R	Cuneus_R
2	PreCG_R	Precentral_R	47	LING_L	Lingual_L
3	SFGdor_L	Frontal_Sup_L	48	LING_R	Lingual_R
4	SFGdor_R	Frontal_Sup_R	49	SOG_L	Occipital_Sup_L
5	ORBsup_L	Frontal_Sup_Orb_L	50	SOG_R	Occipital_Sup_R
6	ORBsup_R	Frontal_Sup_Orb_R	51	MOG_L	Occipital_Mid_L
7	MFG_L	Frontal_Mid_L	52	MOG_R	Occipital_Mid_R
8	MFG_R	Frontal_Mid_R	53	IOG_L	Occipital_Inf_L
9	ORBmid_L	Frontal_Mid_Orb_L	54	IOG_R	Occipital_Inf_R
10	ORBmid_R	Frontal_Mid_Orb_R	55	FFG_L	Fusiform_L
11	IFGoperc_L	Frontal_Inf_Oper_L	56	FFG_R	Fusiform_R
12	IFGoperc_R	Frontal_Inf_Oper_R	57	PoCG_L	Postcentral_L
13	IFGtriang_L	Frontal_Inf_Tri_L	58	PoCG_R	Postcentral_R
14	IFGtriang_R	Frontal_Inf_Tri_R	59	SPG_L	Parietal_Sup_L
15	ORBinf_L	Frontal_Inf_Orb_L	60	SPG_R	Parietal_Sup_R
16	ORBinf_R	Frontal_Inf_Orb_R	61	IPL_L	Parietal_Inf_L
17	ROL_L	Rolandic_Oper_L	62	IPL_R	Parietal_Inf_R
18	ROL_R	Rolandic_Oper_R	63	SMG_L	SupraMarginal_L
19	SMA_L	Supp_Motor_Area_L	64	SMG_R	SupraMarginal_R
20	SMA_R	Supp_Motor_Area_R	65	ANG_L	Angular_L
21	OLF_L	Olfactory_L	66	ANG_R	Angular_R
22	OLF_R	Olfactory_R	67	PCUN_L	Precuneus_L
23	SFGmed_L	Frontal_Sup_Medial_L	68	PCUN_R	Precuneus_R
24	SFGmed_R	Frontal_Sup_Medial_R	69	PCL_L	Paracentral_Lobule_L
25	ORBsubmed_L	Frontal_Med_Orb_L	70	PCL_R	Paracentral_Lobule_R
26	ORBsubmed_R	Frontal_Med_Orb_R	71	CAU_L	Caudate_L
27	REC_L	Rectus_L	72	CAU_R	Caudate_R
28	REC_R	Rectus_R	73	PUT_L	Putamen_L
29	INS_L	Insula_L	74	PUT_R	Putamen_R
30	INS_R	Insula_R	75	PAL_L	Pallidum_L
31	ACG_L	Cingulum_Ant_L	76	PAL_R	Pallidum_R
32	ACG_R	Cingulum_Ant_R	77	THA_L	Thalamus_L
33	DCG_L	Cingulum_Mid_L	78	THA_R	Thalamus_R
34	DCG_R	Cingulum_Mid_R	79	HES_L	Heschl_L
35	PCG_L	Cingulum_Post_L	80	HES_R	Heschl_R
36	PCG_R	Cingulum_Post_R	81	STG_L	Temporal_Sup_L
37	HIP_L	Hippocampus_L	82	STG_R	Temporal_Sup_R
38	HIP_R	Hippocampus_R	83	TPOsup_L	Temporal_Pole_Sup_L
39	PHG_L	ParaHippocampal_L	84	TPOsup_R	Temporal_Pole_Sup_R
40	PHG_R	ParaHippocampal_R	85	MTG_L	Temporal_Mid_L
41	AMYG_L	Amygdala_L	86	MTG_R	Temporal_Mid_R
42	AMYG_R	Amygdala_R	87	TPOmid_L	Temporal_Pole_Mid_L
43	CAL_L	Calcarine_L	88	TPOmid_R	Temporal_Pole_Mid_R
44	CAL_R	Calcarine_R	89	ITG_L	Temporal_Inf_L
45	CUN_L	Cuneus_L	90	ITG_R	Temporal_Inf_R

Table S4. The detailed brain region names of the BN atlas (https://atlas.brainnetome.org/).

Lobe	Gyrus	Left and Right Hemisphere	Label	Label	Anatomical and Modified Cyto-
**Frontal Lobe**	SFG, Superior Frontal Gyrus	SFG_L(R)_7_1	1	2	*A8m, medial area 8*
		SFG_L(R)_7_2	3	4	*A8dl, dorsolateral area 8*
		SFG_L(R)_7_3	5	6	*A9l, lateral area 9*
		SFG_L(R)_7_4	7	8	*A6dl, dorsolateral area 6*
		SFG_L(R)_7_5	9	10	*A6m, medial area 6*
		SFG_L(R)_7_6	11	12	*A9m,medial area 9*
		SFG_L(R)_7_7	13	14	*A10m, medial area 10*
	MFG, Middle Frontal Gyrus	MFG_L(R)_7_1	15	16	*A9/46d, dorsal area 9/46*
		MFG_L(R)_7_2	17	18	*IFJ, inferior frontal junction*
		MFG_L(R)_7_3	19	20	*A46, area 46*
		MFG_L(R)_7_4	21	22	*A9/46v, ventral area 9/46*
		MFG_L(R)_7_5	23	24	*A8vl, ventrolateral area 8*
		MFG_L(R)_7_6	25	26	*A6vl, ventrolateral area 6*
		MFG_L(R)_7_7	27	28	*A10l, lateral area10*
	IFG, Inferior Frontal Gyrus	IFG_L(R)_6_1	29	30	*A44d,dorsal area 44*
		IFG_L(R)_6_2	31	32	*IFS, inferior frontal sulcus*
		IFG_L(R)_6_3	33	34	*A45c, caudal area 45*
		IFG_L(R)_6_4	35	36	*A45r, rostral area 45*
		IFG_L(R)_6_5	37	38	*A44op, opercular area 44*
		IFG_L(R)_6_6	39	40	*A44v, ventral area 44*
	OrG, Orbital Gyrus	OrG_L(R)_6_1	41	42	*A14m, medial area 14*
		OrG_L(R)_6_2	43	44	*A12/47o, orbital area 12/47*
		OrG_L(R)_6_3	45	46	*A11l, lateral area 11*
		OrG_L(R)_6_4	47	48	*A11m, medial area 11*
		OrG_L(R)_6_5	49	50	*A13, area 13*
		OrG_L(R)_6_6	51	52	*A12/47l, lateral area 12/47*
	PrG, Precentral Gyrus	PrG_L(R)_6_1	53	54	*A4hf, area 4(head and face region)*
		PrG_L(R)_6_2	55	56	*A6cdl, caudal dorsolateral area 6*
		PrG_L(R)_6_3	57	58	*A4ul, area 4 (upper limb region)*
		PrG_L(R)_6_4	59	60	*A4t, area 4 (trunk region)*
		PrG_L(R)_6_5	61	62	*A4tl, area 4 (tongue and larynx region)*
		PrG_L(R)_6_6	63	64	*A6cvl, caudal ventrolateral area 6*
	PCL, Paracentral Lobule	PCL_L(R)_2_1	65	66	*A1/2/3ll, area1/2/3 (lower limb region)*
		PCL_L(R)_2_2	67	68	*A4ll, area 4, (lower limb region)*
**Temporal Lobe**	STG, Superior Temporal Gyrus	STG_L(R)_6_1	69	70	*A38m, medial area 38*
		STG_L(R)_6_2	71	72	*A41/42, area 41/42*
		STG_L(R)_6_3	73	74	*TE1.0 and TE1.2*
		STG_L(R)_6_4	75	76	*A22c, caudal area 22*
		STG_L(R)_6_5	77	78	*A38l, lateral area 38*
		STG_L(R)_6_6	79	80	*A22r, rostral area 22*
	MTG, Middle Temporal Gyrus	MTG_L(R)_4_1	81	82	*A21c, caudal area 21*
		MTG_L(R)_4_2	83	84	*A21r, rostral area 21*
		MTG_L(R)_4_3	85	86	*A37dl, dorsolateral area37*
		MTG_L(R)_4_4	87	88	*aSTS, anterior superior temporal sulcus*
	ITG, Inferior Temporal Gyrus	ITG_L(R)_7_1	89	90	*A20iv, intermediate ventral area 20*
		ITG_L(R)_7_2	91	92	*A37elv, extreme lateroventral area37*
		ITG_L(R)_7_3	93	94	*A20r, rostral area 20*
		ITG_L(R)_7_4	95	96	*A20il, intermediate lateral area 20*
		ITG_L(R)_7_5	97	98	*A37vl, ventrolateral area 37*
		ITG_L(R)_7_6	99	100	*A20cl, caudolateral of area 20*
		ITG_L(R)_7_7	101	102	*A20cv, caudoventral of area 20*
	FuG, Fusiform Gyrus	FuG_L(R)_3_1	103	104	*A20rv, rostroventral area 20*
		FuG_L(R)_3_2	105	106	*A37mv, medioventral area37*
		FuG_L(R)_3_3	107	108	*A37lv, lateroventral area37*
	PhG, Parahippocampal	PhG_L(R)_6_1	109	110	*A35/36r, rostral area 35/36*
		PhG_L(R)_6_2	111	112	*A35/36c, caudal area 35/36*

	Gyrus	PhG_L(R)_6_3	113	114	*TL, area TL (lateral PPHC, posterior*
		PhG_L(R)_6_4	115	116	*A28/34, area 28/34 (EC, entorhinal*
		PhG_L(R)_6_5	117	118	*TI, area TI (temporal agranular insular*
		PhG_L(R)_6_6	119	120	*TH, area TH (medial PPHC)*
	pSTS, posterior Superior	pSTS_L(R)_2_1	121	122	*rpSTS, rostroposterior superior temporal*
		pSTS_L(R)_2_2	123	124	*cpSTS, caudoposterior superior temporal*
**Parietal Lobe**	SPL, Superior Parietal Lobule	SPL_L(R)_5_1	125	126	*A7r, rostral area 7*
		SPL_L(R)_5_2	127	128	*A7c, caudal area 7*
		SPL_L(R)_5_3	129	130	*A5l, lateral area 5*
		SPL_L(R)_5_4	131	132	*A7pc, postcentral area 7*
		SPL_L(R)_5_5	133	134	*A7ip, intraparietal area 7(hIP3)*
	IPL, Inferior Parietal Lobule	IPL_L(R)_6_1	135	136	*A39c, caudal area 39(PGp)*
		IPL_L(R)_6_2	137	138	*A39rd, rostrodorsal area 39(Hip3)*
		IPL_L(R)_6_3	139	140	*A40rd, rostrodorsal area 40(PFt)*
		IPL_L(R)_6_4	141	142	*A40c, caudal area 40 (PFm)*
		IPL_L(R)_6_5	143	144	*A39rv, rostroventral area 39 (PGa)*
		IPL_L(R)_6_6	145	146	*A40rv, rostroventral area 40 (PFop)*
	Pcun, Precuneus	PCun_L(R)_4_1	147	148	*A7m, medial area 7 (PEp)*
		PCun_L(R)_4_2	149	150	*A5m, medial area 5 (PEm)*
		PCun_L(R)_4_3	151	152	*dmPOS, dorsomedial parietooccipital*
		PCun_L(R)_4_4	153	154	*A31, area 31 (Lc1)*
	PoG, Postcentral Gyrus	PoG_L(R)_4_1	155	156	*A1/2/3ulhf, area 1/2/3(upper limb, head*
		PoG_L(R)_4_2	157	158	*A1/2/3tonIa, area 1/2/3(tongue and*
		PoG_L(R)_4_3	159	160	*A2, area 2*
		PoG_L(R)_4_4	161	162	*A1/2/3tru, area1/2/3(trunk region)*
**Insular Lobe**	INS, Insular Gyrus	INS_L(R)_6_1	163	164	*G, hypergranular insula*
		INS_L(R)_6_2	165	166	*vIa, ventral agranular insula*
		INS_L(R)_6_3	167	168	*dIa, dorsal agranular insula*
		INS_L(R)_6_4	169	170	*vId/vIg, ventral dysgranular and granular*
		INS_L(R)_6_5	171	172	*dIg, dorsal granular insula*
		INS_L(R)_6_6	173	174	*dId, dorsal dysgranular insula*
**Limbic Lobe**	CG, Cingulate Gyrus	CG_L(R)_7_1	175	176	*A23d, dorsal area 23*
		CG_L(R)_7_2	177	178	*A24rv, rostroventral area 24*
		CG_L(R)_7_3	179	180	*A32p, pregenual area 32*
		CG_L(R)_7_4	181	182	*A23v, ventral area 23*
		CG_L(R)_7_5	183	184	*A24cd, caudodorsal area 24*
		CG_L(R)_7_6	185	186	*A23c, caudal area 23*
		CG_L(R)_7_7	187	188	*A32sg, subgenual area 32*
**Occipital Lobe**	MVOcC, MedioVentral Occipital Cortex	MVOcC _L(R)_5_1	189	190	*cLinG, caudal lingual gyrus*
		MVOcC _L(R)_5_2	191	192	*rCunG, rostral cuneus gyrus*
		MVOcC _L(R)_5_3	193	194	*cCunG, caudal cuneus gyrus*
		MVOcC _L(R)_5_4	195	196	*rLinG, rostral lingual gyrus*
		MVOcC _L(R)_5_5	197	198	*vmPOS, ventromedial parietooccipital*
	LOcC, lateral Occipital Cortex	LOcC_L(R)_4_1	199	200	*mOccG, middle occipital gyrus*
		LOcC _L(R)_4_2	201	202	*V5/MT+, area V5/MT+*
		LOcC _L(R)_4_3	203	204	*OPC, occipital polar cortex*
		LOcC_L(R)_4_4	205	206	*iOccG, inferior occipital gyrus*
		LOcC _L(R)_2_1	207	208	*msOccG, medial superior occipital gyrus*
		LOcC _L(R)_2_2	209	210	*lsOccG, lateral superior occipital gyrus*
**Subcortical Nuclei**	Amyg, Amygdala	Amyg_L(R)_2_1	211	212	*mAmyg, medial amygdala*
		Amyg_L(R)_2_2	213	214	*lAmyg, lateral amygdala*
	Hipp, Hippocampus	Hipp_L(R)_2_1	215	216	*rHipp, rostral hippocampus*
		Hipp_L(R)_2_2	217	218	*cHipp, caudal hippocampus*
	BG, Basal Ganglia	BG_L(R)_6_1	219	220	*vCa, ventral caudate*
		BG_L(R)_6_2	221	222	*GP, globus pallidus*
		BG_L(R)_6_3	223	224	*NAC, nucleus accumbens*
		BG_L(R)_6_4	225	226	*vmPu, ventromedial putamen*
		BG_L(R)_6_5	227	228	*dCa, dorsal caudate*
		BG_L(R)_6_6	229	230	*dlPu, dorsolateral putamen*
	Tha, Thalamus	Tha_L(R)_8_1	231	232	*mPFtha, medial prefrontal thalamus*
		Tha_L(R)_8_2	233	234	*mPMtha, premotor thalamus*

		Tha_L(R)_8_3	235	236	*Stha, sensory thalamus*
		Tha_L(R)_8_4	237	238	*rTtha, rostral temporal thalamus*
		Tha_L(R)_8_5	239	240	*PPtha, posterior parietal thalamus*
		Tha_L(R)_8_6	241	242	*Otha, occipital thalamus*
		Tha_L(R)_8_7	243	244	*cTtha, caudal temporal thalamus*
		Tha_L(R)_8_8	245	246	*lPFtha, lateral prefrontal thalamus*

References

Aerts HJ, Velazquez ER, Leijenaar RT, Parmar C, Grossmann P, Carvalho S*, et al.* Decoding tumour phenotype by noninvasive imaging using a quantitative radiomics approach. Nat Commun 2014; 5: 4006.
Feng F, Wang P, Zhao K, Zhou B, Yao H, Meng Q*, et al.* Radiomic Features of Hippocampal Subregions in Alzheimer's Disease and Amnestic Mild Cognitive Impairment. Front Aging Neurosci 2018; 10: 290.
He Y, Chen ZJ, Evans AC. Small-world anatomical networks in the human brain revealed by cortical thickness from MRI. Cereb Cortex 2007; 17(10): 2407-19.
Kim HJ, Shin JH, Han CE, Kim HJ, Na DL, Seo SW*, et al.* Using Individualized Brain Network for Analyzing Structural Covariance of the Cerebral Cortex in Alzheimer's Patients. Front Neurosci 2016; 10: 394.
Seidlitz J, Vasa F, Shinn M, Romero-Garcia R, Whitaker KJ, Vertes PE*, et al.* Morphometric Similarity Networks Detect Microscale Cortical Organization and Predict Inter-Individual Cognitive Variation. Neuron 2018; 97(1): 231-47 e7.
Tijms BM, Series P, Willshaw DJ, Lawrie SM. Similarity-based extraction of individual networks from gray matter MRI scans. Cereb Cortex 2012; 22(7): 1530-41.
Zhao K, Ding YH, Han Y, Fan Y, Alexander-Bloch AF, Han T*, et al.* Independent and reproducible hippocampal radiomic biomarkers for multisite Alzheimer's disease: diagnosis, longitudinal progress and biological basis. Science Bulletin 2020; 65(13): 1103-13.
